# Progressive multiple sclerosis patients show substantial lesion activity that correlates with clinical disease severity and sex: a retrospective autopsy cohort analysis

**DOI:** 10.1007/s00401-018-1818-y

**Published:** 2018-02-13

**Authors:** Sabina Luchetti, Nina L. Fransen, Corbert G. van Eden, Valeria Ramaglia, Matthew Mason, Inge Huitinga

**Affiliations:** 1 Laboratory of Neuroimmunology, Netherlands Institute for Neuroscience (NIN), an Institute of the Royal Netherlands Academy of Arts and Sciences, Meibergdreef 47, 1105 BA Amsterdam, The Netherlands; 2 Department of Immunology, University of Toronto, 1 King’s College Circle, Toronto, ON M5S 1A8 Canada

**Keywords:** Multiple sclerosis, Neuropathology, Lesion activity, Sex characteristics, Chronic active lesions, Remyelination

## Abstract

Multiple sclerosis (MS) is a highly heterogeneous disease with large inter-individual differences in disease course. MS lesion pathology shows considerable heterogeneity in localization, cellular content and degree of demyelination between patients. In this study, we investigated pathological correlates of disease course in MS using the autopsy cohort of the Netherlands Brain Bank (NBB), containing 182 MS brain donors. Using a standardized autopsy procedure including systematic dissection from standard locations, 3188 tissue blocks containing 7562 MS lesions were dissected. Unbiased measurements of lesion load were made using the tissue from standard locations. Lesion demyelinating and innate inflammatory activity were visualized by immunohistochemistry for proteolipid protein and human leukocyte antigen. Lesions were classified into active, mixed active/inactive (also known as chronic active), inactive or remyelinated, while microglia/macrophage morphology was classified as ramified, amoeboid or foamy. The severity score was calculated from the time from first symptoms to EDSS-6. Lesion type prevalence and microglia/macrophage morphology were analyzed in relation to clinical course, disease severity, lesion load and sex, and in relation to each other. This analysis shows for the first time that (1) in progressive MS, with a mean disease duration of 28.6 ± 13.3 years (mean ± SD), there is substantial inflammatory lesion activity at time to death. 57% of all lesions were either active or mixed active/inactive and 78% of all patients had a mixed active/inactive lesion present; (2) patients that had a more severe disease course show a higher proportion of mixed active/inactive lesions (*p* = 6e−06) and a higher lesion load (*p* = 2e−04) at the time of death, (3) patients with a progressive disease course show a higher lesion load (*p* = 0.001), and a lower proportion of remyelinated lesions (*p* = 0.03) compared to patients with a relapsing disease course, (4) males have a higher incidence of cortical grey matter lesions (*p* = 0.027) and a higher proportion of mixed active/inactive lesions compared to females across the whole cohort (*p* = 0.007). We confirm that there is a higher proportion of mixed active/inactive lesions (*p* = 0.006) in progressive MS compared to relapsing disease. Identification of mixed active/inactive lesions on MRI is necessary to determine whether they can be used as a prognostic tool in living MS patients.

**Electronic supplementary material:**

The online version of this article (10.1007/s00401-018-1818-y) contains supplementary material, which is available to authorized users.

## Introduction

Multiple sclerosis (MS) is a chronic inflammatory disease of the central nervous system, which is characterized by demyelination, inflammation and neuroaxonal damage [27, 39]. It is a heterogeneous disease with large inter-individual differences in clinical presentation [32], radiological appearance of the lesions [17] and response to immunomodulatory therapy [18] as well as sex differences [23].

In line with the broad clinical spectrum of MS, the pathological manifestation of the disease is also highly heterogeneous between patients and has been at the centre of investigation for decades [12, 13, 33, 36]. In biopsy tissue from patients with MS, four immunopathological patterns of MS lesions have been identified based on the presence of immunoglobulin deposits, active complement or evidence of primary oligodendrocyte pathology [33, 36]. However, in a post-mortem cohort of chronic MS patients, white matter lesions showed a homogenous pattern of antibody and complement-mediated demyelination [6]. Although lesion activity in the chronic late phase of MS does not show the four different patterns, large inter-individual differences in inflammatory and demyelinating activity, meningeal inflammation, degree of axonal loss, and remyelination are still observed in post-mortem MS cohort studies [9, 13, 35, 38]. The pathological and clinical heterogeneity of MS suggest that different pathogenic mechanisms may be at work, with the significant implication that MS patients may require individualized therapeutic approaches. However, pathological correlates for clinical disease course, disease severity and sex differences in MS are currently not well explored [13]. This is probably due to the fact that world-wide, the availability of clinically and pathologically well-characterized MS brain tissue collections of sufficient power is limited.

In this study, we analyzed the heterogeneity in MS lesion activity in relation to clinical disease course, disease severity and sex in the MS autopsy cohort of the Netherlands Brain Bank (NBB), consisting of 182 clinically well-documented MS brain donors. In total, 3188 tissue blocks were analyzed containing 7562 lesions. Based on previously proposed criteria, MS lesions in white and cortical grey matter were categorized according to location, degree of demyelination, and innate inflammatory activity as determined by microglia/macrophage presence and morphology [25, 29, 44]. Our data show for the first time that demyelinating and innate inflammatory lesion activity is substantial at time of death in progressive MS patients with long-standing disease and that clinical disease course, disease severity and sex strongly correlate with MS lesion characteristics in autopsy tissue.

## Materials and methods

### Subjects

The cohort comprises 182 MS donors that came to autopsy between 1990 and 2015 in the framework of the NBB. Informed consent was given by the donors for brain autopsy and for the use of material and clinical data for research purposes, in compliance with national ethical guidelines. MS pathology was confirmed by a certified neuropathologist (Prof. J. M. Rozemuller or Prof. P. van der Valk, VUmc, Amsterdam, The Netherlands). The clinical diagnosis of MS was confirmed for all patients, and the clinical course was defined as relapsing (8 relapsing remitting and 6 progressive relapsing patients), secondary progressive (SP) or primary progressive (PP) by a neurologist (Prof C. H. Polman, VU Medical Center, Amsterdam or Dr. S. Luchetti, Netherlands Institute for Neuroscience, Amsterdam) according to McDonald or Poser criteria. Clinical course was not determined in 12 patients. Patients with clinical or pathological features of acute disseminated encephalomyelitis or neuromyelitis optica or confounding CNS pathologies such as large ischemic, hemorrhagic lesions or cerebral metastasis were excluded. Disability status was determined by retrospective chart analysis using Kurtzke’s Expanded Disability Status Scale (EDSS) and the time from first symptoms to EDSS-6 and EDSS-8 was determined. In 14 patients, information about the time to EDSS-6 was unavailable. The clinical characteristics of the MS cohort of the NBB are summarized in Table 1. No significant differences were found in age at death, post-mortem delay, post-mortem pH and year of autopsy between sexes or disease course types.

**Table 1:** Clinical characteristics of multiple sclerosis patients

	Total	Female	Male	PP	SP	Relapsing
Number of patients^a^	182	113	69	56 (F33/M23)	100 (F61/M39)	14 (F10/M4)
Duration of disease, years (mean ± SD)	28.6 ± 13.3	29.2 ± 13.5	27.5 ± 12.8	27.6 ± 11.7	29.9 ± 14.2	24.2 ± 11.6
Time to EDSS-6, years (mean ± SD)	16.3 ± 11.7	16.9 ± 11.8	15.2 ± 11.7	14.0 ± 10.2	17.9 ± 12.7	11.8 ± 4.9
Age at death, years (mean ± SD)	62.0 ± 15.4	63.6 ± 15.6	59.5 ± 14.9	65.3 ± 13.2	59.8 ± 16.5	60.8 ± 15.2
Post-mortem delay, hours (mean ± SD)	9.3 ± 6.9	9.1 ± 6.8	9.7 ± 7.1	8.7 ± 4.4	8.9 ± 5.4	11.6 ± 13.7
Year of autopsy (mean ± range)	2005 (1990–2015)	2004 (1990–2015)	2005 (1991–2015)	2005 (1993–2015)	2005 (1990–2015)	2007 (1997–2012)
Number of tissue blocks dissected per donor (mean ± SD	18 ± 11	17 ± 11	19 ± 10	18 ± 9.9	18 ± 11	19 ± 9.6
Number of supratentorial tissue blocks dissected per donor (mean ± SD)	12 ± 6.9	11 ± 7.4	12 ± 6.2	11 ± 6.6	12 ± 7.2	11 ± 6.4
Cause of death (*n*)						
Euthanasia	39					
Respiratory failure/pneumonia	58					
Cardiovascular failure	16					
Sepsis	13					
Cachexia	9					
Suicide	2					
Other^b^	20					
Not reported	25					

*PP* primary progressive, *SP* secondary progressive, relapsing, *F* female, *M* male

^a^Clinical course was not available for 12 patients

^b^i.e., dehydration, ileus, gastrointestinal bleeding, liver insufficiency, multi-organ failure, surgery complication, lung embolism

### Tissue dissection

The NBB autopsy procedures were approved by the Ethical Committee of the VU University Medical Center in Amsterdam, the Netherlands. Blocks were dissected at seven standardized locations from the brainstem (BRS) and eight standardized locations from the spinal cord (SPC), two cervical, two thoracic, two lumbar, two sacral, from 161 and 120 patients, respectively. Visible MS plaques (PLA) were dissected during autopsy from 150 patients. In addition, since 2001 (116 patients), MS lesions were also dissected on post-mortem MRI guidance (MRI) on 1-cm-thick coronal brain slices cut throughout the brain [15]. Tissue dissection characteristics are shown in suppl. Table 1 (Online Resource 1). Similar numbers of tissue blocks (18 ± 11 per patient, from which 12 ± 6.9 were from supratentorial locations) were characterized in all clinical patient groups.

### Characterization of MS lesions

For each patient, all available archived material was analyzed. Double immunostaining was performed on sections from all dissected tissue blocks to visualize proteolipid protein (PLP) (MCA839G, AbD Serotec, Oxford, UK, with DAB) and human leukocyte antigen (HLA-DR-DQ, referred to as HLA) (M0775, CR3/43, DAKO, Denmark, with DAB-nickel), as previously described [14, 20, 19, 34]. White matter, cortical grey matter and spinal cord MS lesions were characterized according to the system of van der Valk et al. and Kuhlmann et al. [25, 44] Innate inflammatory lesion activity was determined as presence of HLA+ microglia/macrophages. We used microglia/macrophage morphology to quantify phagocytosing activity since amoeboid and foamy microglia/macrophages are thought to be actively phagocytosing myelin [5, 29].

Reactive sites and four types of white matter lesions were discriminated based on demyelination and presence of HLA+ microglia/macrophages, while cortical grey matter lesions, identified as demyelinated areas, were classified by location [4]. The criteria for both are given in Table 2 and Fig. 9. For active and mixed active/inactive lesions, microglia/macrophage morphology was scored: 0 = thin and ramified; 0.5 = amoeboid with few ramification; 1 = foamy. The Microglial/Macrophage activation Score (MMAS) was calculated as the average of these scores for each patient.

**Table 2:** Definition of lesion-scoring system and overall occurrence

Lesion type	Definition	Score	No. lesions	% of total lesions	% patients with lesion type present
Reactive site	No demyelination, aggregates of HLA+ microglia/macrophages	1	770		64.8 (118/182)
*White matter*					
Active	Demyelination, HLA+ microglia/macrophages throughout the lesion	2	1357	23.8	70.9 (129/182)
Mixed active/inactive (same as chronic active in Kuhlmann, Lassmann, and Brück [24], includes smoldering in Frischer et al. [13])	Demyelination, hypocellular and gliotic centre, accumulation of HLA+ microglia/macrophages at the lesion border	3	1873	32.8	77.5 (141/182)
Inactive	Demyelination, hypocellular and gliotic throughout the lesion	4	1561	27.3	86.8 (158/182)
Remyelinated	Partial myelination, sparse HLA + microglia/macrophages	6	919	16.1	67.6 (123/182)
Total		2/3/4/6	5710	100	96.7 (176/182)
*Cortical grey matter*		5			
Leukocortical (mixed grey-white matter)		I	570	31.3	65.0 (104/160)
Intracortical		II	782	42.2	68.1 (109/160)
Subpial		III	457	24.7	43.1 (69/160)
Subpial extending up to the white matter border		IV	43	2.3	10.6 (17/160)
Total		I/II/III/IV	1852	100	79.4 (127/160)

**Fig. 1:**
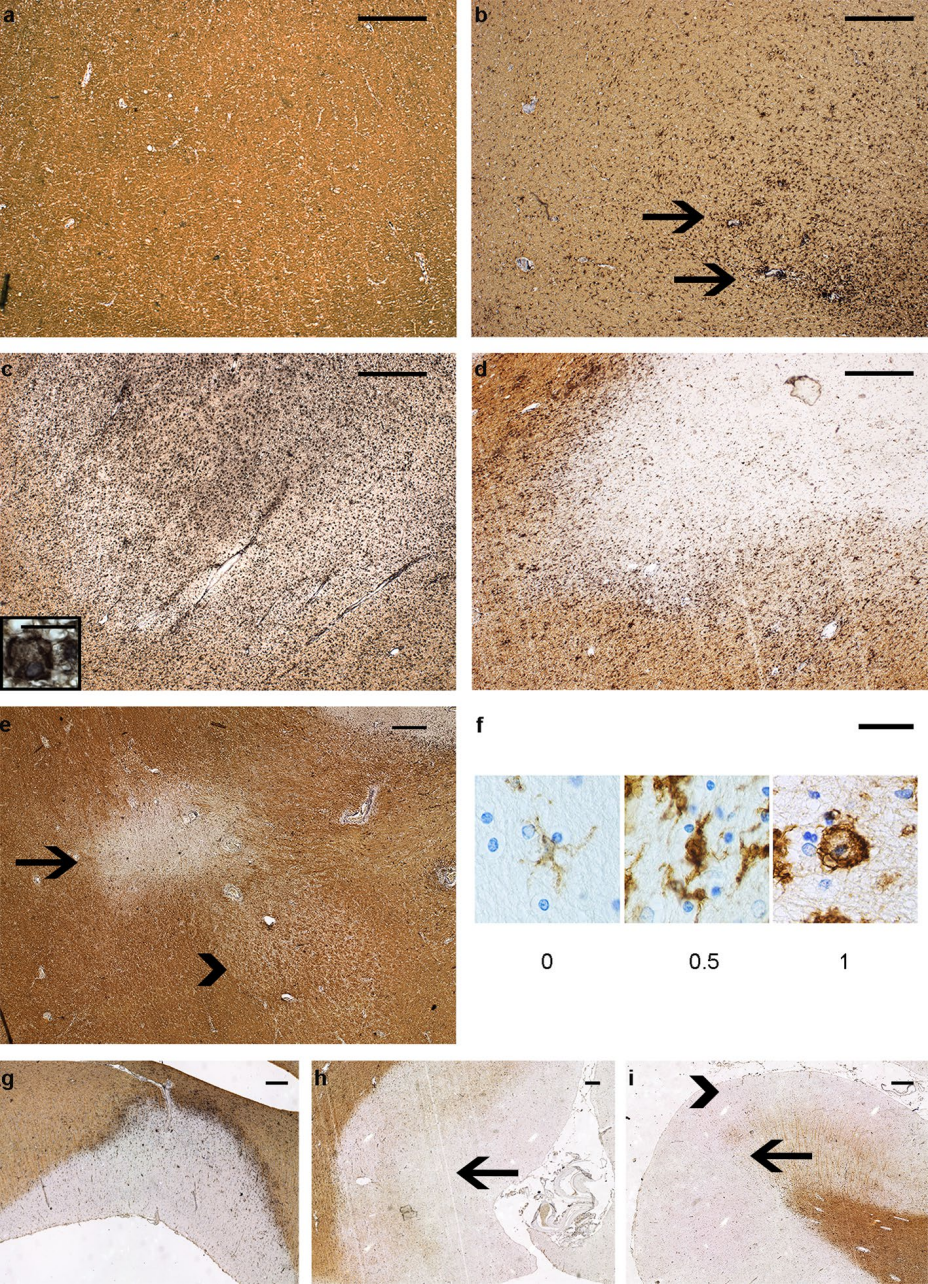
Scoring MS lesion subtypes. Double immunostaining for human leukocyte antigen (HLA, in black), detecting microglia/macrophages, and proteolipid protein (PLP, in brown), detecting myelin on tissue samples from MS patients. **a**–**e** white matter lesions. **a** Normal-appearing white matter (NAWM). **b** Reactive site. **c** Active lesion, with foamy microglia/macrophages. **d** Mixed active/inactive (chronic active) lesion with rounded microglia/macrophages. **e** Inactive lesion (arrow) and inactive remyelinated lesion (arrowhead). **f** Microglia/macrophage morphology score used for all active (2) and mixed active/inactive (3) lesions. 0: Ramified 0.5: Amoeboid 1: Foamy. **g**–**i** Cortical grey matter lesions, **g**: leukocortical lesion (I), **h**: intracortical lesion (II, arrow). **i** Subpial lesions (III, arrowhead and IV arrow). Scale bar **a**–**e** and **g**–**i** 0.5 mm. Scale bar **f** 0.025 mm. Scale bar inset in **c**: 0.020 mm

In previous studies [13], active lesions were further divided into early and late active lesions using the presence of minor myelin proteins within microglia/macrophages. To provide more insight in the presence of myelin oligodendrocyte glycoprotein (MOG) in microglia/macrophages in active lesions, co-localization of IBA-1 and MOG is measured in a subset of active (*n* = 5) lesions dissected at a standard location at the level of the medulla oblongata. Double sequential immunostaining of MOG (1:200 Abcam, EP4281) with AEC (red), followed by antigen retrieval and then IBA-1 (1:3000 WAKO cat.no. 019-19741) with NBT-BCIP (blue) was performed including controls showing no MOG staining after antigen retrieval. Co-localization was measured using spectral microscopy [30], shown in suppl. Figure 2 (Online Resource 3). Examples of this classification system and lesion scoring are shown in Figs. 1, 9 and suppl. Figure 1 (Online Resource 2).

### Lesion load and proportions of lesion subtype calculations

For each tissue block dissected, all visibly distinct lesions were classified separately as shown in suppl. Figure 1 (Online Resource 2). To avoid sampling bias that may arise when identifying lesions by MRI or macroscopically, lesion load was determined by counting MS lesions in brainstem tissue blocks, as these were dissected at standard locations. One section per block was used and all lesions in the section that were visibly distinct were counted separately. Reactive sites were excluded since it remains unclear whether these represent the initial stage of an MS lesion [22]. Reactive site load was calculated separately as counts of reactive sites present in the brainstem blocks. Where lesions appeared to contain mixed features (e.g., mixed microglial/macrophage morphology) the most preponderant type was recorded. Cortical grey matter lesions were scored as either present or absent, cortical tissue blocks were available for 160 donors. The relative proportions of lesion subtypes were calculated from all lesions scored in both standard locations (BRS and SPC) and PLA and MRI tissue blocks. The number of patients with specific block types available are shown in suppl. Table 1 (Online Resource 1). The calculation of the proportions of lesion subtypes is defined in Table 3.

**Table 3:** Definition of pathological parameters used for analysis

	Definition	Tissue blocks used for calculations
Lesion load	Counts of all active, mixed active/inactive, inactive and remyelinated lesions	Standardly dissected brainstem tissue blocks (BRS)
Reactive load	Counts of all reactive sites	Standardly dissected brainstem tissue blocks (BRS)
Cortical grey matter lesion presence	Yes/no present	In all supratentorial blocks (PLA + MRI) with cortex
Proportion of active lesions	Active/(active + mixed active/inactive + inactive + remyelinated)	From all tissue blocks dissected
Proportion of mixed active/inactive (chronic active) lesions	Chronic active/(active + mixed active/inactive + inactive + remyelinated)	From all tissue blocks dissected from a donor
Proportion remyelinated lesions	Remyelinated/(inactive + remyelinated)	From all tissue blocks dissected from a donor
Microglia/macrophage activity score (for active and mixed active/inactive lesions)	Average for each patient Ramified = 0 Amoeboid = 0.5 Foamy = 1	From all tissue blocks dissected from a donor

### Statistical analysis

Disease severity score was calculated as 5-log (years to EDSS-6 +1), giving a score between 1 (least severe) and 5 (most severe). Lesion load and reactive site load were transformed as log(*x* + 1). Correlations between severity score, lesion load, and proportions of lesion subtypes were tested using Pearson’s product-moment correlation coefficient. Differences in severity scores, lesion load, reactive site load and lesion subtype proportions were tested against sex, presence of mixed active/inactive lesions and presence of cortical grey matter lesions using *t* tests with unequal variance (n of each group was > 30 in these tests). Differences in severity score, lesion load and reactive site load were tested against MS types using Kruskall–Wallis and Wilcoxon tests (non-parametric tests were used here because the relapsing group contained < 25 patients). Proportions of lesion subtypes and the MMAS were tested against MS clinical types and against location using quasibinomial-generalized linear models (GLM) and Tukey’s post hoc tests. Associations among sex, MS subtypes and presence/absence of cortical grey matter lesions were tested using Fisher’s exact test. Correlations with severity score, lesion load and differences between MS subtypes were also tested with linear models including sex as a factor, which showed that sex does not influence these outcomes. All analyses were carried out in the statistical computing environment R.

## Results

### The MS cohort of the Netherlands Brain Bank consists mainly of patients with (primary or secondary) progressive disease

In 182 MS patients that came to autopsy at the NBB 3188 tissue blocks are dissected and characterized containing 7562 lesions. The clinical disease course of this cohort consists of relapsing (8%), PP (31%) and SP (55%) patients with mean disease duration of 24.2 ± 11.6, 27.6 ± 11.7 and 29.9 ± 14.2 years, respectively, and the overall mean disease duration of 28.6 ± 13.3 years (range 2–64 years), indicating that this cohort consists mainly of chronic progressive MS patients (Table 1).

### MS lesion activity is substantial at time of death in long-term progressive disease

Notably, in the whole NBB cohort, fifty-seven percent (57%) of demyelinated white matter and mixed grey–white matter lesions were either active or mixed active/inactive (chronic active), showing that there is considerable inflammatory lesion activity even in autopsy patients with long-term progressive disease (Table 2). Furthermore, even in patients with the longest disease duration (42–64 years), active or mixed active/inactive lesions account for 34% of all lesions, further supporting the notion that lesion activity is substantial in the progressive late phase of the disease. Proportions of lesion subtypes versus years to EDSS-6, years to EDSS-8 and disease duration are shown in suppl. Figure 3 (Online Resource 4).

### Lesion load and proportion of mixed active/inactive lesions positively correlates with disease severity

**Fig. 2:**
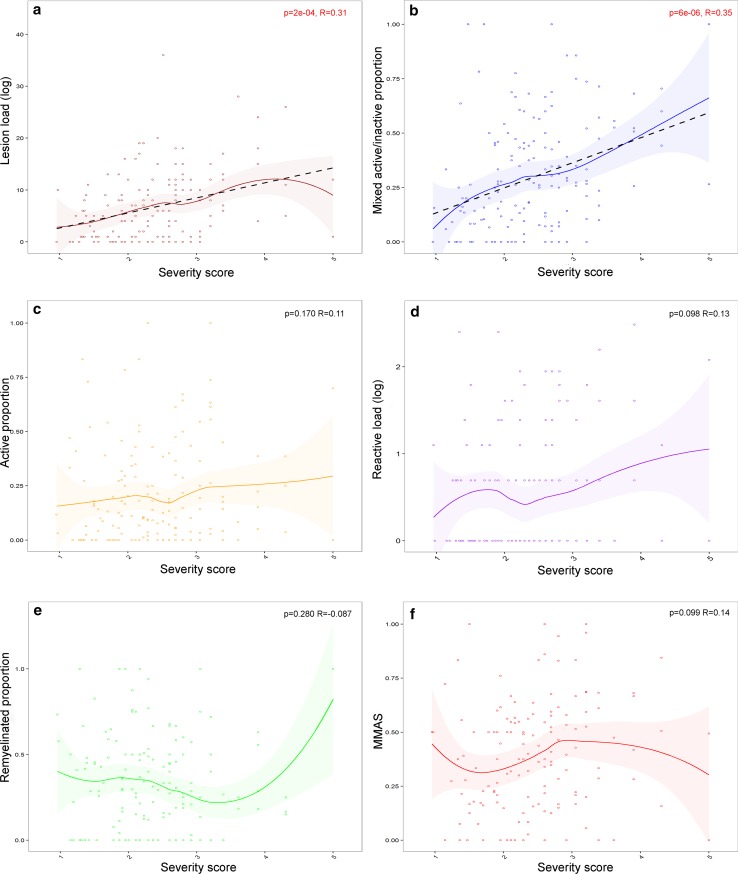
Relationship between lesion activity and clinical disease severity. Patients with a more severe disease course have a higher lesion load (**a**) and a higher proportion of mixed active/inactive (chronic active) lesions (**b**) than patients with a less severe disease course. The disease severity did not correlate with the proportion of active lesions (**c**), the reactive load (**d**), the proportion of remyelinated lesions (**e**) or the MMAS (**f**). Severity score is calculated as 5-log (years to EDSS-6 +1), with shorter time to EDSS-6 the patient has a higher severity score. The relationship between each parameter and severity score is shown as a loess-smoothed fit with 95% confidence intervals. Where a significant correlation was found, the straight-line fit is shown (black-dotted line). Pearson’s product-moment correlation coefficient was used

Patients with more severe disease (shorter time to EDSS-6) had a higher lesion load (*p* = 2e−04, *R* = 0.31), and a higher proportion of mixed active/inactive lesions (*p* = 6e−06, *R* = 0.35) (Fig. 2). No significant correlation was found between the disease severity score and the proportions of active lesions (*p* = 0.17, *R* = 0.11) or remyelinated lesions (*p* = 0.28, *R* = − 0.087, note a single outlier causes a non-significant increase in remyelinated lesions in the most severe patients), or with reactive site load (*p* = 0.10, *R* = 0.14), or microglia/macrophage activity score (*p* = 0.10, *R* = 0.14) (Fig. 2).

**Fig. 3:**
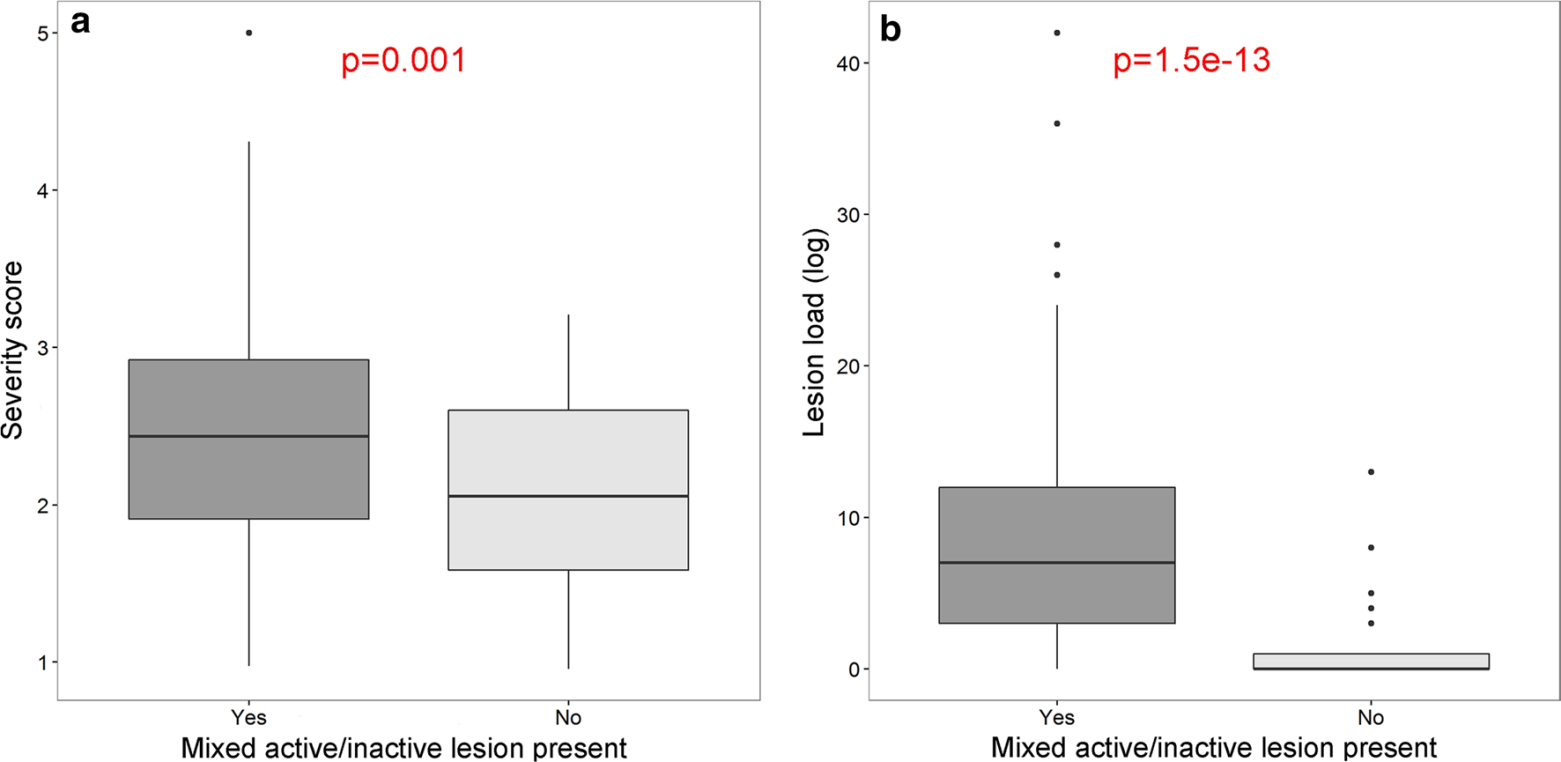
Mixed active/inactive lesion presence relates to severity and lesion load. Patients with a mixed active/inactive (chronic active) lesion present (*n* = 141) have a more severe disease course (**a**) and a higher lesion load (**b**) compared to patients without a mixed active/inactive lesion present (*n* = 41). *t* test with unequal variances was used

In 22.5% of MS patients, no mixed active/inactive lesions were observed, and these patients had a less severe disease course (*p* = 0.001) and lower lesion load (*p* = 1.5E−13) compared to patients where mixed active/inactive lesions were present (Table 2 and Fig. 3).

In suppl. Figure 3 (Online Resource 4), we show all lesion type proportions versus years to EDSS-6, years to EDSS-8 and disease duration. Regardless of the measure of disease severity, patients with more severe disease (shorter time to EDSS-6 or EDSS-8 or shorter disease duration) had relatively more mixed active/inactive lesions, a higher proportion of lesions with foamy microglia/macrophages, and relatively fewer remyelinated lesions. The proportion of active lesions is a constant low percentage (range 16–27%) in all disease severity and disease duration groups.

Cortical grey matter lesions were observed in 79.4% of patients (Table 2) and disease severity did not differ significantly between patients with or without cortical grey matter lesions (*p* = 0.53).

### SP and PP patients have a higher lesion load with a higher proportion of mixed active/inactive and lower proportion of remyelinated lesions compared to relapsing patients

**Fig. 4:**
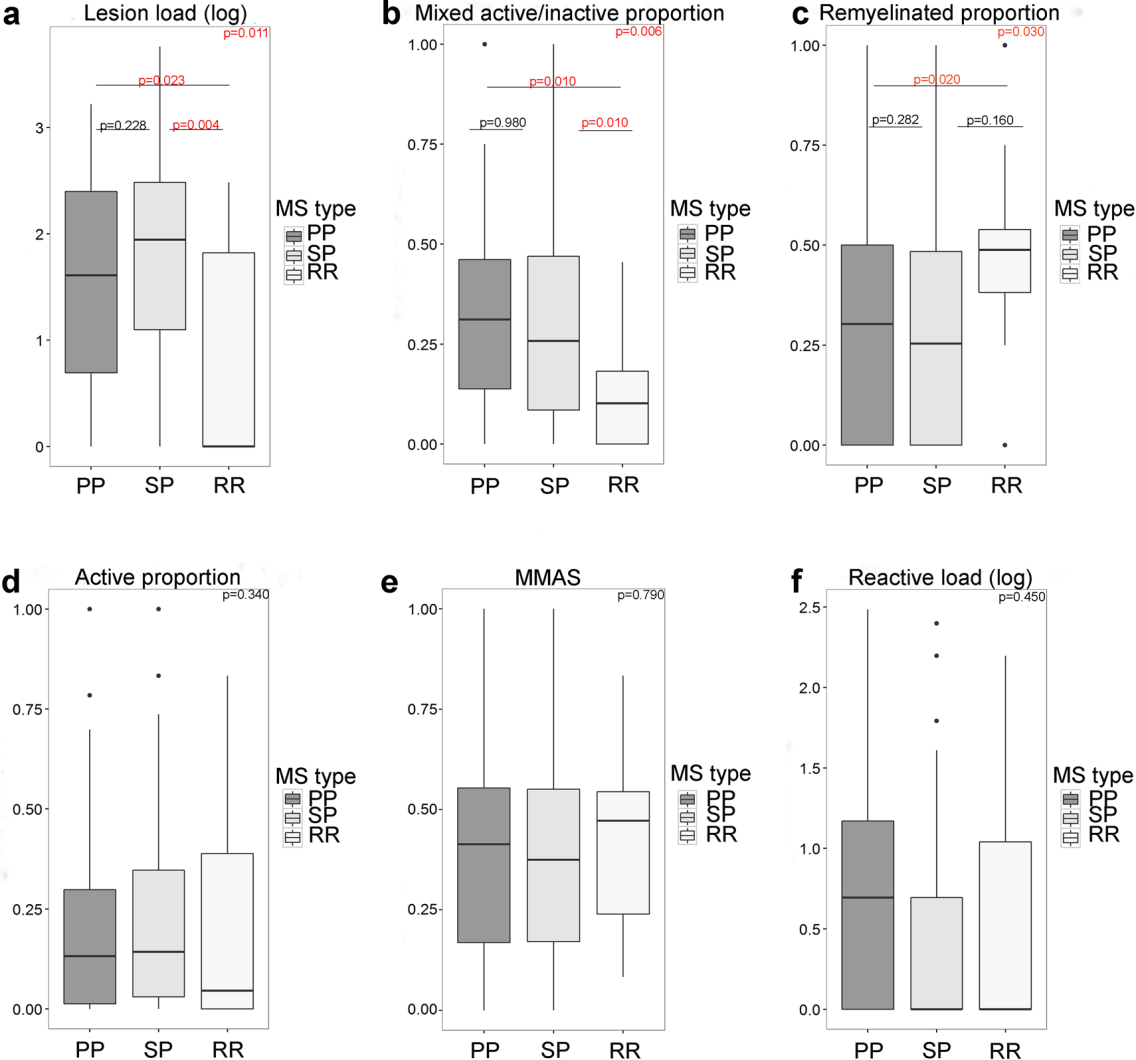
Lesion activity in clinical MS types. Relapsing patients have a significantly lower lesion load (**a**), a lower proportion of mixed active/inactive (chronic active) lesions (**b**) and a higher proportion of remyelinated lesions (**c**) as compared to PP and SP MS. The proportion of active lesions (**d**) MMAS (**e**) and reactive site load (**f**) were not significantly different between the clinical MS types. Lesion load and reactive load were transformed as log(*x* + 1). The Kruskall–Wallis and Wilcoxon tests of pairs were used for lesion load and reactive load. For lesion subtype proportions and MMAS, a quasibinomial-generalized linear model was used with Tukey’s post hoc tests

SP (*n* = 93) and PP (*n* = 52) patients did not differ significantly in lesion load, reactive site load or in proportions of lesion subtypes (Fig. 4). However, compared to relapsing (*n* = 14) patients, both SP and PP patients showed a higher lesion load (Kruskall–Wallis; *p* = 0.011, Wilcoxon: SP vs PP *p* = 0.29 SP vs relapsing *p* = 0.004, PP vs relapsing *p* = 0.023), a higher proportion of mixed active/inactive lesions (GLM *p* = 0.006, Tukey’s post hoc tests SP vs PP *p* = 0.98, SP vs relapsing *p* = 0.01, PP vs relapsing *p* = 0.01) and PP patients had a lower proportion of remyelinated lesions (GLM *p* = 0.03, Tukey’s post hoc tests SP vs PP *p* = 0.28, SP vs relapsing *p* = 0.16, PP vs relapsing *p* = 0.02). No significant differences were found in reactive site load (Kruskall–Wallis, *p* = 0.18), proportion of active lesions (GLM, *p* = 0.34) or microglia/macrophage activity score (GLM, *p* = 0.79). No difference in presence of cortical grey matter lesions was found between Relapsing, PP or SP MS (*p* = 0.29).

### Males have a higher proportion of mixed active/inactive lesions and a higher incidence of cortical grey matter lesions compared to females

**Fig. 5:**
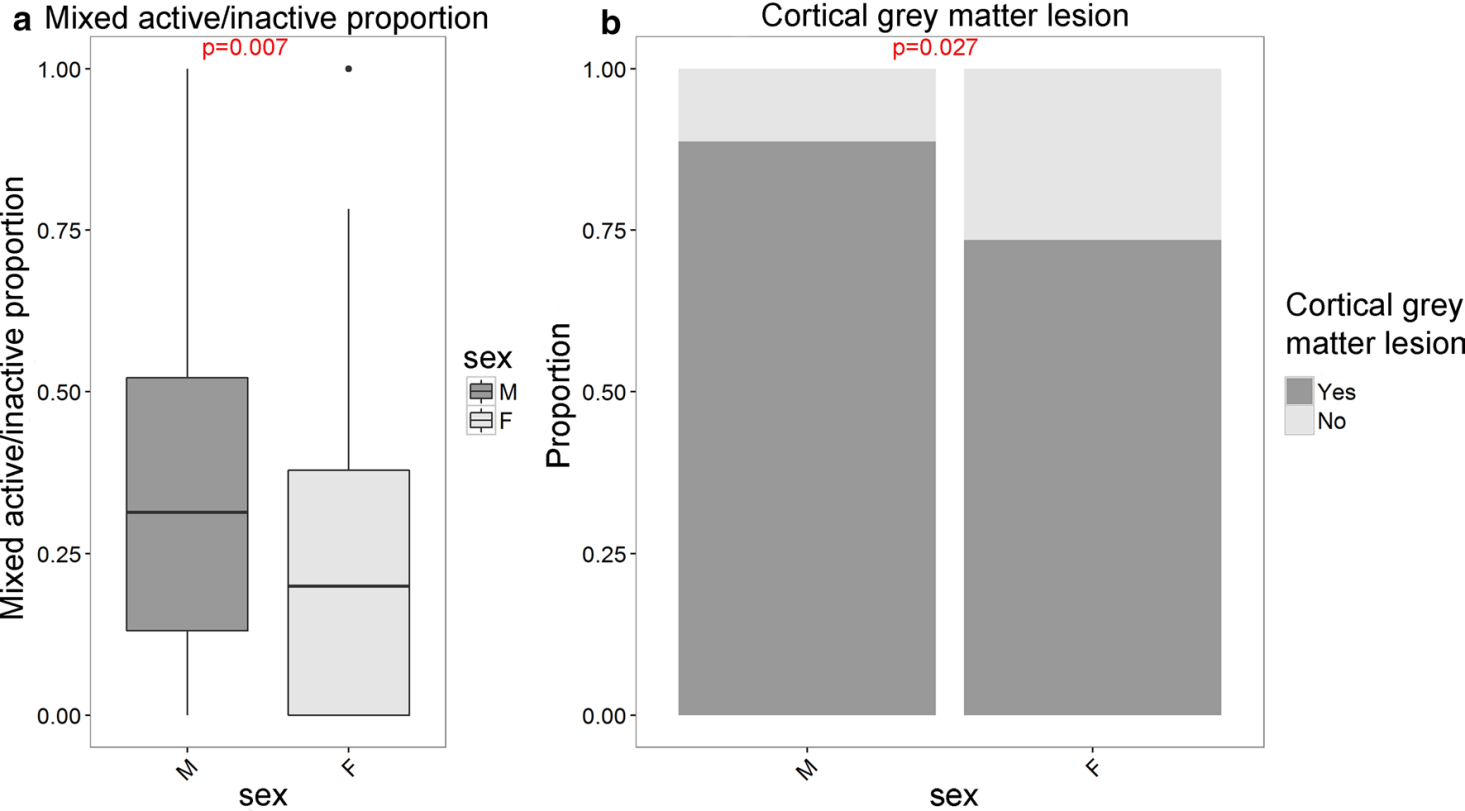
Sex differences in MS lesion activity and cortical grey matter lesions. Males have a higher proportion of mixed active/inactive (chronic active) lesions (**a**, *t* test with unequal variances was used) and a higher incidence of cortical grey matter lesions (**b**, Fisher’s exact test) as compared to females

Males had a higher proportion of mixed active/inactive lesions than females (*p* = 0.007). Males had a higher incidence of cortical grey matter lesions (*p* = 0.027) (Fig. 5). An inverse relationship between cortical grey matter lesion presence and severity score was found in females but not in males [suppl. Figure 5a, (Online Resource 6)] (GLM, females *p* = 0.004, males *p* = 0.56). Consistent with this, females with cortical grey matter lesions present have a higher disease severity score than females without (*p* = 0.004), while in males there is no difference (*p* = 0.73) [suppl. Figure 5b, (Online Resource 6)].

### Lesion load correlates with the proportions of lesion subtypes

**Fig. 6:**
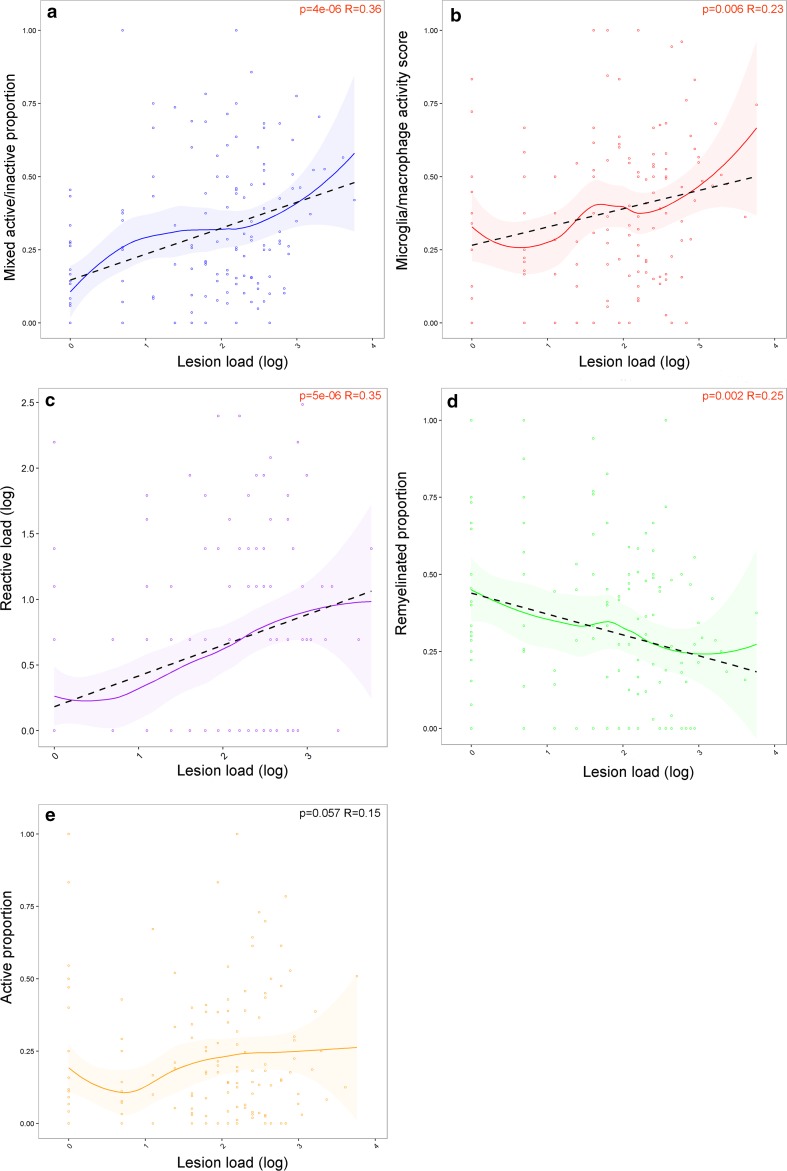
The relationship between lesion load and lesion activity. The lesion load positively correlates with the proportion of mixed active/inactive (chronic active) lesions (**a**), MMAS (**b**), and reactive load (**c**), whereas it negatively correlates with the proportion of remyelinated lesions (**d**). The proportion of active lesions (**e**) did not significantly correlate with lesion load. The relationship between each parameter and lesion load is shown as a loess-smoothed fit with 95% confidence intervals. Where a significant correlation was found, the straight-line fit is shown (black-dotted line). Pearson’s product-moment correlation coefficient was used

Lesion load correlated positively with proportion of mixed active/inactive lesions (*p* = 4e−06, *R* = 0.36), microglia/macrophage activity score (*p* = 0.006 *R* = 0.23) and reactive site load (*p* = 5e−06, *R* = 0.35) whereas it correlated negatively with proportion of remyelinated lesions (*p* = 0.002, *R* = − 0.25). No significant correlation was found with the proportion of active lesions (*p* = 0.057, *R* = 0.15) (Fig. 6).

### The presence of cortical grey matter lesions relates to lesion load and to the proportions of lesion subtypes in white matter

**Fig. 7:**
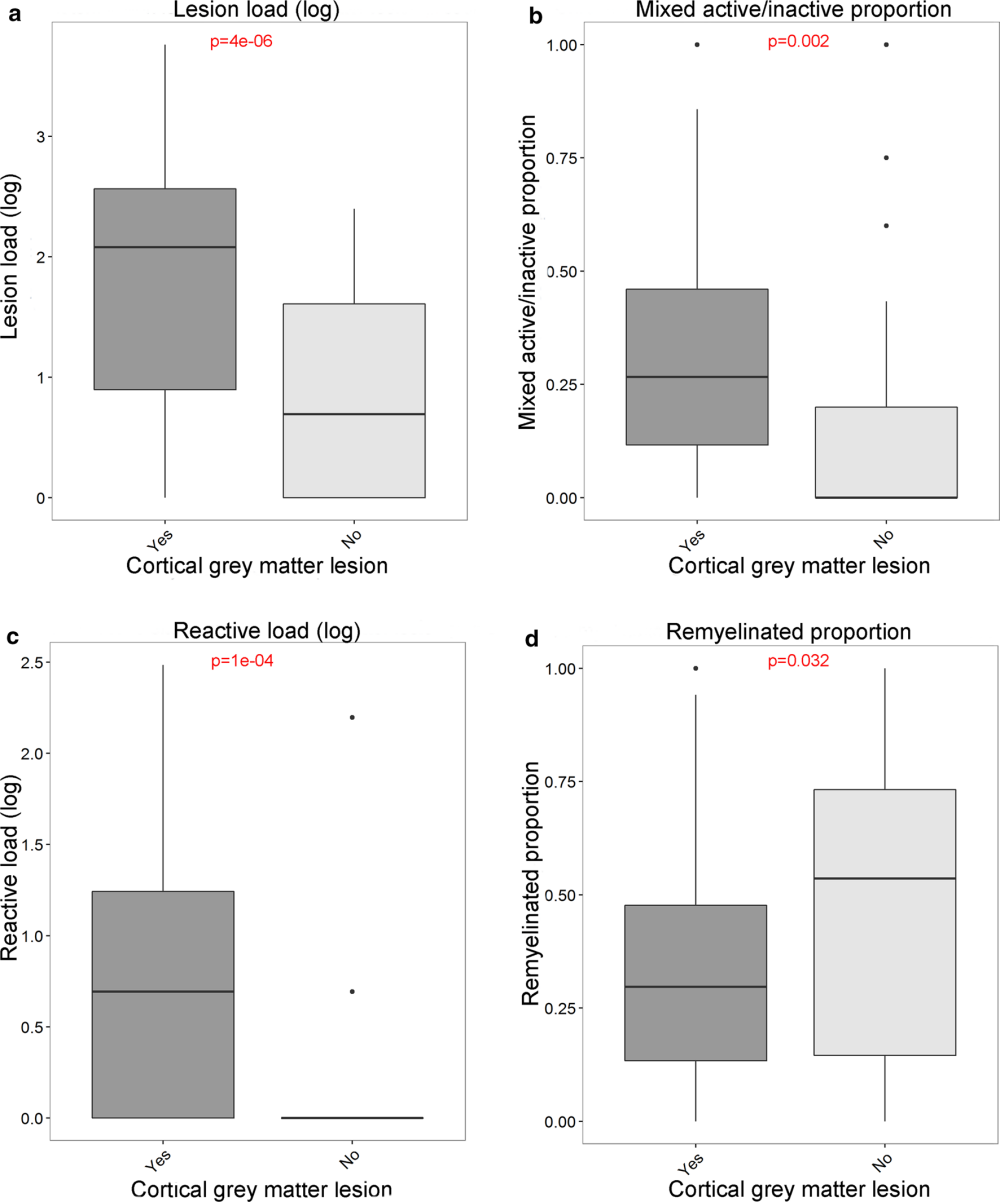
The relationship between the presence of cortical grey matter lesions and white matter lesion activity. Patients with cortical grey matter lesions have a significantly higher lesion load (**a**), higher reactive load (**c**), higher proportion of mixed active/inactive (chronic active) lesions (**b**) and a lower proportion of remyelinated lesions (**d**). Lesion load and reactive load were determined in standardly dissected brainstem tissue blocks and proportions of chronic active and remyelinated lesions were determined in white matter. *t* test with unequal variance was used

Patients with cortical grey matter lesions had a higher lesion load (*p* = 4e−06), a higher proportion of mixed active/inactive lesions (*p* = 0.002) and a higher reactive site load (*p* = 2e−04) but a lower proportion of remyelinated lesions (*p* = 0.032). No relation was found between the presence of cortical grey matter lesions and the proportion of active lesions (*p* = 0.28) or the microglia/macrophage activity score (*p* = 0.13) (Fig. 7).

The correlation of cortical grey matter lesions with lesion load, proportion of mixed active/inactive lesions, reactive site load and proportion of remyelinated lesions, was strongest in type I (leukocortical) (*p* = 3e−08, *p* = 0.013, *p* = 9e−05, *p* = 0.002, respectively) and II (intracortical) (*p* = 5e−05, *p* = 0.003, *p* = 0.023, *p* = 0.025, respectively) lesions. Subpial III and IV lesions showed a weaker relation with lesion load (*p* = 0.003) and no relation with mixed active/inactive (chronic active) lesions (*p* = 0.075), reactive sites (*p* = 0.16) and remyelinated lesions (*p* = 0.17) [suppl. Figure 6 (Online Resource 7)].

### Relationships between lesion subtypes

**Fig. 8:**
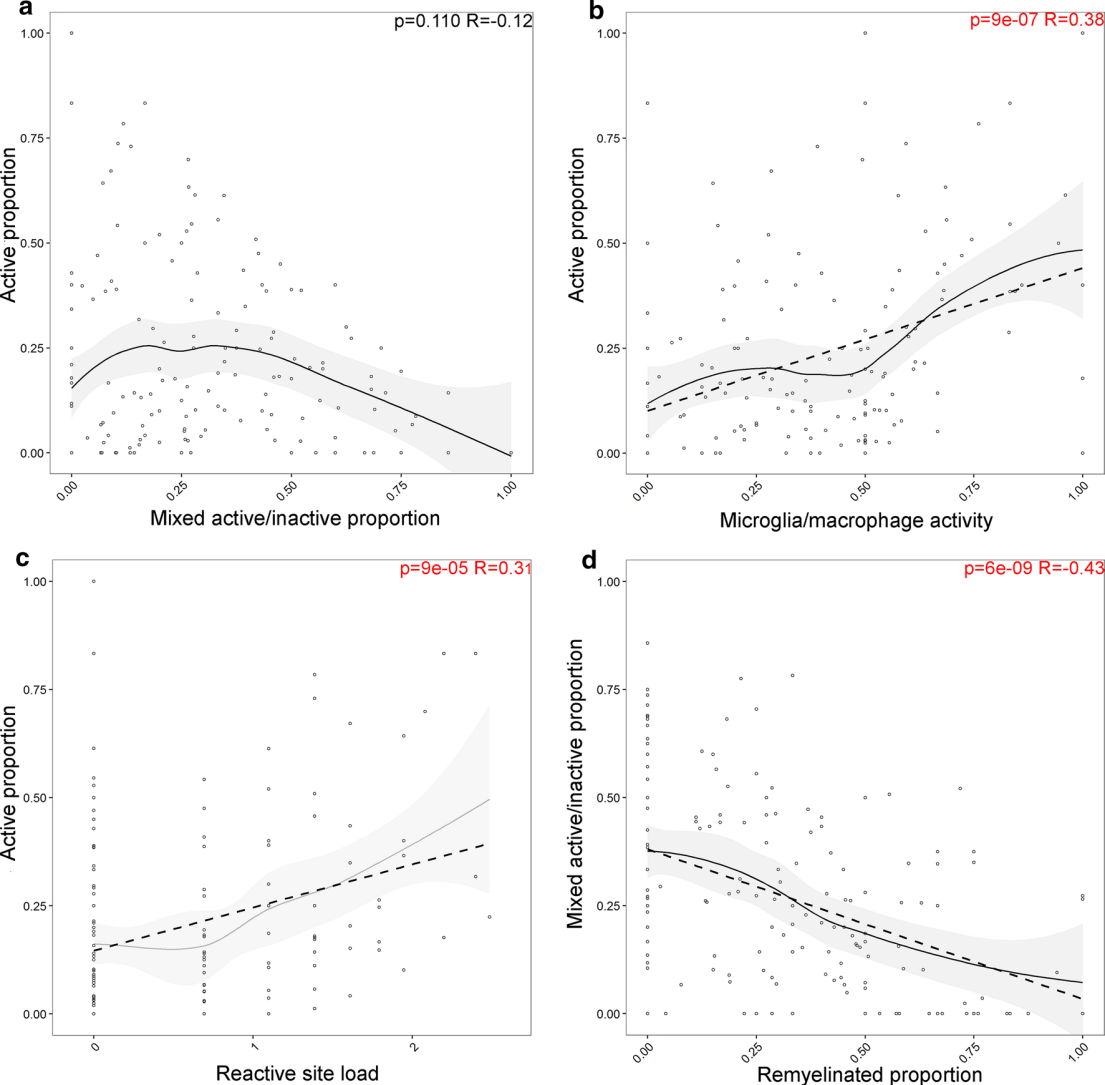
The relationship between active lesion proportion and mixed active/inactive lesion proportion with lesion activity. The proportion of active lesions significantly correlated with the MMAS (**a**) and the reactive site load (log transformed) (**b**). There is no relationship between the proportion of active lesions and mixed active/inactive (chronic active) lesions (**c**), whereas the proportion of mixed active/inactive lesions inversely correlated with the proportion of remyelinated lesions (**d**). Pearson’s product-moment correlation coefficient was used

The proportion of active lesions is not significantly correlated with the proportion of mixed active/inactive lesions (*p* = 0.11, *R* = − 0.12) (Fig. 8). The proportion of active lesions positively correlated with the microglia/macrophage activity score (*p* = 9e−07, *R* = 0.38) and reactive site load (*p* = 9e−05, *R* = 0.31), while the proportion of mixed active/inactive lesions does not show these correlations. However, the mixed active/inactive proportion inversely correlated with the proportion of remyelinated lesions (*p* = 6e−09, *R* = − 0.43).

### Lesion activity across central nervous system locations

The proportion of mixed active/inactive lesions was significantly lower in spinal cord than in brainstem (*p* = 1e−8). The proportion of remyelinated lesions (22% vs 4%, *p* = 5e−05) was significantly higher in SPC as compared to the BRS. The distribution of lesion subtypes in different tissue samples (SPC, BRS and PLA/MRI samples) is shown in suppl. Figure 7 (Online Resource 8).

### Minor myelin protein in microglia/macrophages in active lesions

In a subset of active and mixed active/inactive lesions dissected at a standard location at the level of the medulla oblongata (active *n* = 5 and mixed active/inactive *n* = 12) microglia/macrophages showed in variable amount co-localization with MOG. For active lesions, mean percentage of IBA-1 signal that colocalized with MOG signal was 13.6% (range 1.4–39.2%), whereas for the mixed active/inactive lesions this was 8.8% (range 0.02–46.3) [suppl. Figure 2 (Online Resource 3)].

## Discussion

This study demonstrates that the lesion characteristics in post-mortem MS autopsy tissue correlate with clinical disease severity and sex in an extensive collection of MS autopsy patients and highly powered analysis. Lesion inflammatory and demyelinating activity is widely present even in the late stage of disease (mean disease duration of 29 years), with 57% of the 7562 lesions examined being either active or mixed active/inactive (chronic active) at the time of death. This contrasts with previous observations that inflammatory disease processes decline in long-term disease [13].

**Fig. 9:**
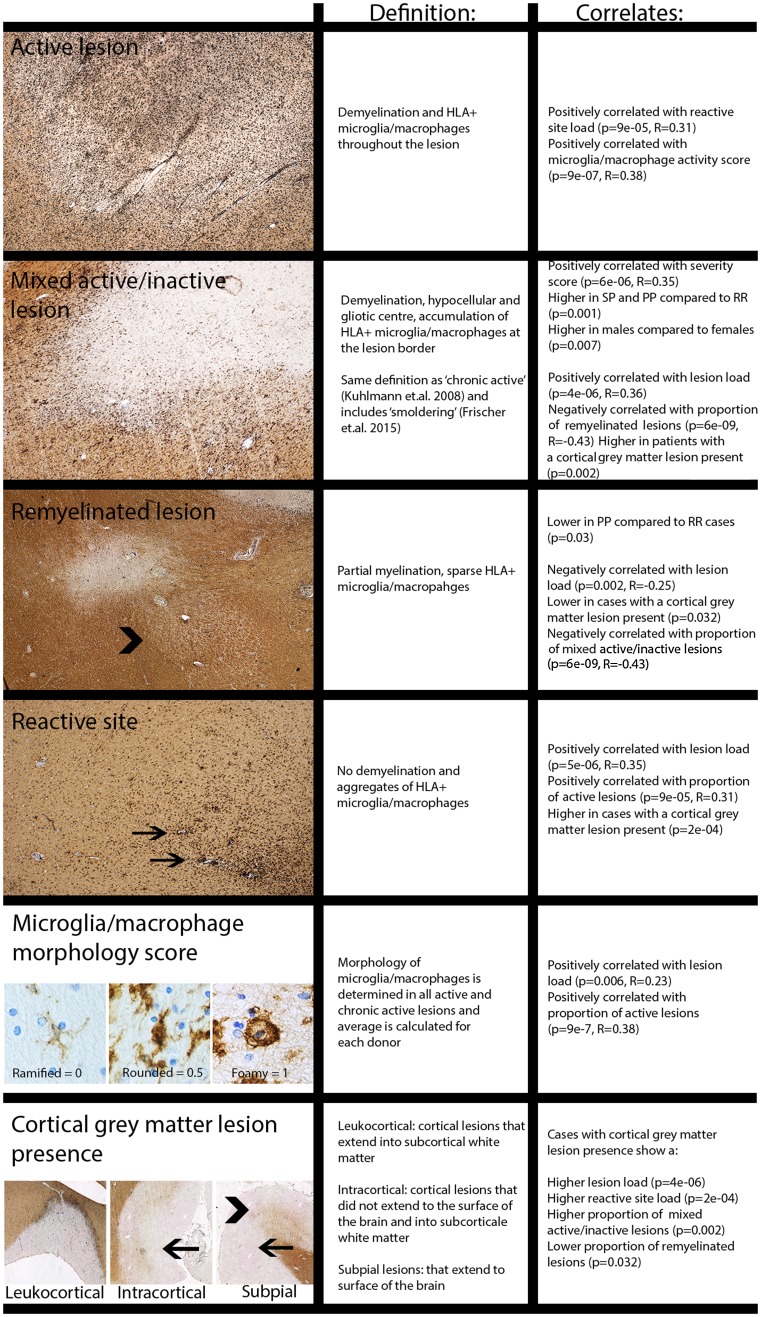
Overview of lesion activity scoring and correlates shown in analysis. Examples are shown of each subtype of white and grey matter lesions, and of microglial/macrophage morphology scoring. Alongside are shown their definitions and significant correlations

This study also shows for the first time that: (1) MS brain donors with shorter time to EDSS-6 have a higher lesion load and a higher proportion of mixed active/inactive lesions at time of death. (2) MS patients with a progressive disease course have a higher lesion load and a lower proportion of remyelinated lesions compared to relapsing MS patients. We confirm that the proportion of mixed active/inactive lesions are increased in progressive compared to relapsing patients [13]. PP and SP patients show comparable lesion activity. (3) Males have a higher incidence of cortical grey matter lesions compared to females. We show for the first time that males have a higher proportion of mixed active/inactive lesions for the whole cohort, where this was previously shown only for one age group [13]. (4) The lesion load is positively correlated with the proportion of mixed active/inactive lesions, reactive sites, microglia/macrophages activity and inversely with remyelinated lesions, using a systematic unbiased histological quantification of lesion load by analyzing standardly dissected tissue blocks from the brainstem. (5) The presence of cortical grey matter lesions is related to lesion load, mixed/active inactive lesions, reactive sites and remyelinated lesions by correlating histologically determined presence of cortical grey matter lesions to parameters of lesion activity (summarised in Fig. 9).

The NBB MS autopsy cohort differs somewhat from the general MS patient population, since it contains a higher proportion of PP patients, being 25% compared to 10% in the general population [31]. The relapsing patients in this cohort consist of a combination of eight relapsing remitting (RR) and six progressive relapsing (PR) patients. Compared to the recently described autopsy MS cohort by Frischer et al. [13], the NBB cohort contains MS patients with a longer mean disease duration (29 vs 12 years) and higher proportion of PP and SP patients.

In the cohort of Frischer et al. [13] active lesions, with microglia/macrophages throughout the lesion, were subdivided in early and late active based on the presence of minor myelin proteins within microglia/macrophages. It is suggested that this inclusion of minor myelin proteins reflects recent myelin phagocytosis [25]. Quantification of the inclusion of MOG (a minor myelin protein) within IBA-1-positive cells suggest that 40% of the active lesions show > 20% IBA-1 signal colocalized with MOG signal, therefore, these can be considered early active. This is a comparable incidence with the data of Frischer et al. [13] showing in the 10–30 years disease duration groups that 25–50% of active lesions can be defined as early active.

This analysis shows for the first time that lesion load correlated strongly with disease severity. This is in line with a number of prospective MRI studies [7, 11, 42]. We also show for the first time that relapsing patients have a lower lesion load, and a higher proportion of remyelinated lesions and confirm that they have a lower proportion of mixed active/inactive lesions. This is in line with MRI findings showing lower lesion load in RR compared to SP MS [11, 42]. Our data show no differences in lesion pathology between PP and SP MS patients, in line with the finding that SP and PP patients do not differ in aspects of clinical disease course once they enter the progressive phase of the disease [28].

The proportion of mixed active/inactive lesions (chronic active lesions) is a strong predictor of both disease severity and lesion load. These lesions represent ongoing innate demyelinating and inflammatory activity, and include the lesions described as ‘smouldering’ by Frischer et al. [13]. In that study, ‘smouldering’ lesions were not predominant in very severe patients (disease duration < 1 year), and instead peaked at medium disease duration (around 18 years) before declining. In contrast, we found that the proportion of mixed active/inactive lesions increases monotonically with severity and these lesions are present in all patients with a time to EDSS 6 < 4 years. Similar results are shown with time to EDSS-8 and disease duration. These findings suggest that mixed active/inactive lesions are involved in disease progression, and it is plausible that this is due to the accumulation of this lesion type over time. Indeed, active demyelination in mixed active/inactive lesions has been correlated with axonal injury in these lesions [12, 13] which could explain the progressive MS clinical course. Patients without a mixed active/inactive lesion present had a less severe disease course and lower lesion load. The development of (PET-)MRI techniques (e.g. [8, 21, 10]) that allow the specific identification of these mixed active/inactive lesions in living MS patients is needed to determine the evolution of these lesions over time and if these can be used as a prognostic tool.

The proportion of remyelinated lesions was higher in relapsing patients compared to PP and SP patients. In addition, the proportion of remyelinated lesions inversely correlated with lesion load, with the proportion of mixed active/inactive lesions and with the presence of cortical grey matter lesions. We hypothesize that the increased chronic lesion activity leads to greater axonal loss, resulting in fewer intact but demyelinated axons available for remyelination when lesions subsequently become inactive. The correlation of lesion activity with axonal injury and loss is currently being investigated in the NBB autopsy cohort.

Interestingly, the proportion of active lesions shows no correlation with disease severity and lesion load. Frischer et al. [13] found that the proportion of active lesions was the highest in acute MS patients with very short duration of disease (< 1 year) and declined rapidly with disease duration. In that study, MS patients with a disease duration > 5 years show a constant low percentage of active lesions in all disease duration groups (on average 25%), which is in line with our data. Because there is no correlation between the proportion of active lesions and disease severity, we hypothesize that activated microglia/macrophages present throughout a (partially) demyelinated lesion, can be both involved in demyelination and/or remyelination. In line with this hypothesis, it is very recently shown on PET-MRI that active lesions with microglia/macrophages throughout the lesion are either shrinking or expanding after 1-year follow-up [3].

The active lesion proportion, MMAS and reactive site load all correlate with each other, suggesting that high microglia/macrophage activity is linked to reactive site and active lesion formation. Mixed active/inactive lesions are thought to derive from active lesions, but in our data there is no correlation between mixed active/inactive lesion proportion and either active proportion or reactive site load, leaving open the possibility that mixed active/inactive lesions and active lesions may form independently.

The reactive site load showed a positive correlation with the lesion load, with the proportion of active lesions and with the presence of cortical grey matter lesions, all suggesting that reactive sites are linked to lesion formation. Interestingly, the reactive site load did not correlate with mixed active/inactive lesion proportion. Indeed reactive sites, which comprise clusters of activated microglia/macrophages, have been previously described as ‘pre-active’ lesions and suggested to represent the earliest stage of lesion development [43]. They have been found prevalently around active lesions [22] and they have been associated with axons undergoing Wallerian degeneration [2, 22, 41]. Although these data support the hypothesis that reactive sites may be involved in lesion formation, a recent study showed that microglial clusters are a common hallmark of neuropathology, being present not only in the MS brain but also in non-demyelinating diseases such as stroke [37]. Therefore, it remains to be determined if and how microglial cells arranged in clusters around (partially) myelinated axons in MS develop into a phagocytic state, thereby forming an active demyelinating lesion.

MS patients with presence of an intra-cortical and leuko-cortical grey matter lesion have a higher lesion load, proportion of mixed active/inactive lesions and reactive load and lower proportion of remyelinated lesions. Apparently, demyelinating and inflammatory lesion activity in the white matter correlates with cortical grey matter pathology. Indeed, a recent study [26] showed the importance of CCR2+ monocytes that are present in mixed/active inactive MS lesions [40], in the development of cortical demyelination and disease severity in non-human primates with EAE. Interestingly, the presence of subpial MS lesions does not correlate with activity of white matter MS lesions, and therefore, different pathologic mechanisms may play a role in formation of subpial cortical lesions such as local effects of meningeal inflammation [35]. We show a lower number of subpial lesions than expected based on previous publications [16], however, this is likely because cortex was not sampled from standard locations, and therefore, we do not draw conclusions from the absolute numbers and did not use the cortical lesion load for analysis.

The spinal cord contains a lower proportion of mixed active/inactive lesions compared to the brain stem, which is in line with observations from Frischer et al. [13] In the NBB cohort, the proportion of remyelinated lesions is also significantly higher in spinal cord than in brain stem. These findings indicate that disease processes are less aggressive in spinal cord than in the brainstem.

Male MS patients show a higher proportion of mixed active/inactive lesions, in line with their more severe clinical disease course [23]. These mixed active/inactive lesions are similar to the “smouldering” lesions previously reported to be increased in male MS patients, but only in the 45–55 age group [13]. Males also had a higher incidence of cortical grey matter lesions than females, suggesting females are more resistant to cortical lesion formation. Indeed, females with cortical grey matter MS lesions have a shorter time to EDSS-6, thus have a more aggressive disease. Males were previously found to have more intra-cortical lesions by MRI [1]. Recently, we found sex differences in progesterone signalling, where progesterone receptor and its synthetic enzyme are increased in lesions and peri-lesional normal-appearing white matter (NAWM) in females compared to males, suggesting an important role for progesterone in the sex differences in MS pathology [34].

## Conclusion

There is considerable heterogeneity in MS lesion pathology between patients, and lesion characteristics show strong correlations with clinical course, severity and sex. This study shows for the first time that inflammatory lesion activity is substantial at the time of death in progressive MS patients with long-standing disease. In addition, patients that have had a shorter time to EDSS-6 have a higher lesion load and proportion of mixed active/inactive (chronic active) lesions. Males have a higher cortical grey matter lesion incidence and proportion of mixed active/inactive lesions across the whole cohort. Patients with a progressive disease course have a higher lesion load and less remyelinated lesions compared to relapsing patients. Moreover, we confirm that mixed active/inactive lesions are increased in progressive MS patients. Identification of mixed active/inactive lesions on MRI is necessary to determine whether they can be used as a prognostic tool in living MS patients.

## Electronic supplementary material

Below is the link to the electronic supplementary material.

Supplementary material 1 (PDF 36 kb)

Supplementary material 2 (PDF 125 kb)

Supplementary material 3 (PDF 240 kb)

Supplementary material 4 (PDF 203 kb)

Supplementary material 5 (PDF 181 kb)

Supplementary material 6 (PDF 168 kb)

Supplementary material 7 (PDF 116 kb)
