# Supplementary material for: Progressive multiple sclerosis patients show substantial lesion activity that correlates with clinical disease severity and sex: a retrospective autopsy cohort analysis
Source: Acta Neuropathol. 2018 Feb 13;135(4):511–28. doi: 10.1007/s00401-018-1818-y (PMC5978927; doi:10.1007/s00401-018-1818-y)
Supplement: Supplementary file 1 — Supplementary material 1 (PDF 36 kb) [file 401_2018_1818_MOESM1_ESM.pdf]

Online resource 1:

Supplemental Table 1 Characteristics of tissue blocks examined

Mean number of blocks per case for clinical subtypes and sex and the number of cases that have specific block types available. BRS: diagnostic blocks dissected at 7 standardized locations from the brainstem. SPC: diagnostic blocks dissected at 6 standardized locations from the spinal cord. PLA: macroscopically visible MS lesions dissected. MRI: MS lesions dissected on post-mortem MRI guidance. PLA/MRI with cortex: tissue blocks with cortex present.

**MS type/sex**	**No. blocks per case (mean ± SD)**	**Total no. blocks**
all	18 ±0.8	3188
PP	18 ±1.3	
SP	18 ±1.1	
Relapsing	19 ±2.6	
Females	17 ±1.0	
Males	19 ±1.3	
**Block type**	**No. cases per block type**	**% cases per block type**
BRS	161	88,5
SPC	120	65.9
PLA/MRI	162	89,0
PLA	150	82,4
MRI	116	63,7
PLA/MRI with cortex	160	88,9

Article title: Progressive Multiple Sclerosis patients show substantial lesion activity that correlates with clinical disease severity and sex: a retrospective autopsy cohort analysis

Journal name: Acta Neuropathologica

Author names: Sabina Luchetti# MD PhD, Nina L. Fransen# MD MSc, Corbert G. van Eden PhD, Valeria Ramaglia PhD, Matthew Mason* PhD, Inge Huitinga* PhD

Corresponding author: Inge Huitinga, PhD, Leader Neuroimmunology group Netherlands Institute for Neuroscience, e-mail i.huitinga@nin.knaw.nl,
