# Supplementary material for: Progressive multiple sclerosis patients show substantial lesion activity that correlates with clinical disease severity and sex: a retrospective autopsy cohort analysis
Source: Acta Neuropathol. 2018 Feb 13;135(4):511–28. doi: 10.1007/s00401-018-1818-y (PMC5978927; doi:10.1007/s00401-018-1818-y)
Supplement: Supplementary file 2 — Supplementary material 2 (PDF 125 kb) [file 401_2018_1818_MOESM2_ESM.pdf]

Online resource 2:

Supplemental Figure 1 Double immunostaining for human leukocyte antigen (HLA, in black) and proteolipid protein (PLP, in brown) on MS brain sections from tissue blocks dissected by the Netherlands Brainbank

The examples show the annotation of the scoring system, that identifies in a,one reactive site (1); in b, one mixed active/inactive lesion with foamy microglia/macrophages (3.3); in c, one reactive site (1), one mixed active/inactive lesion (3), one inactive lesion (4) and four shadow plaques (6); in d, one active lesion with amoeboid microglia/macrophages (2.2), one active lesion with foamy microglia/macrophages (2.3), two mixed active/inactive lesions with amoeboid microglia/macrophages (3.2), one leukocortical grey matter lesion (5.I), two intracortical grey matter lesions (5.II), three subpial lesions of which one is more superficial (5.III) and two affect multiple layers of the cortex, reaching the border with the white matter (5.IV).

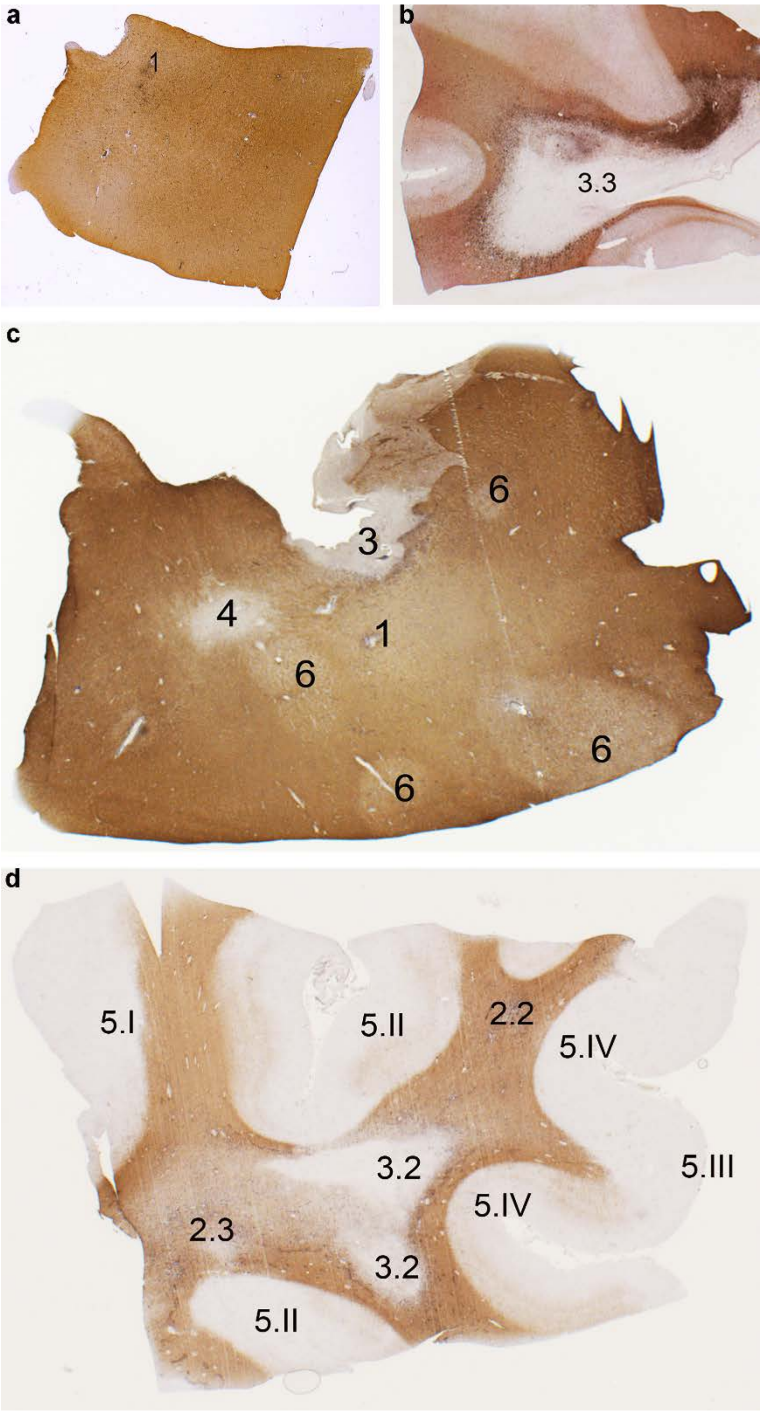

Article title: Progressive Multiple Sclerosis patients show substantial lesion activity that correlates with clinical disease severity and sex: a retrospective autopsy cohort analysis

Journal name: Acta Neuropathologica

Author names: Sabina Luchetti# MD PhD, Nina L. Fransen# MD MSc, Corbert G. van Eden PhD, Valeria Ramaglia PhD, Matthew Mason* PhD, Inge Huitinga* PhD

Corresponding author: Inge Huitinga, PhD, Leader Neuroimmunology group Netherlands Institute for Neuroscience, e-mail i.huitinga@nin.knaw.nl
