# Supplementary material for: Progressive multiple sclerosis patients show substantial lesion activity that correlates with clinical disease severity and sex: a retrospective autopsy cohort analysis
Source: Acta Neuropathol. 2018 Feb 13;135(4):511–28. doi: 10.1007/s00401-018-1818-y (PMC5978927; doi:10.1007/s00401-018-1818-y)
Supplement: Supplementary file 3 — Supplementary material 3 (PDF 240 kb) [file 401_2018_1818_MOESM3_ESM.pdf]

Online resource 3:

Supplemental Fig. 2 Quantification of IBA-1 and MOG co-localization in active and mixed active/inactive lesions.

In 5 active and 12 mixed active/inactive lesions dissected from 13 cases at a standard location of the Medulla Oblongata (b) IBA-1 and MOG double immunohistochemistry was performed using NBT/BCIP (blue) and AEC (red) as chromogens, respectively. After the NBT/BCIP reaction, the sections were boiled for 10 min in microwave 700 watt before incubation with second primary antibody. Control sections showed no MOG signal after boiling the sections (not shown). a. percentages of IBA-1 signal co-localized with the MOG signal in active and mixed active/inactive lesions. b. example of HLA-PLP expression from an active lesion in which 2 regions were analyzed. c. images of a double staining of an active lesion are shown in c and e and composites of spectral images with IBA-1 signal in blue and MOG signal in red are shown in d and f. For each lesion or lesion rim, 2 or 3 pictures were captured and co-localization was measured using the set spectra, the mean % of IBA-1 positive signal co-localized with MOG signal was calculated for each lesion. For the active lesion shown here this is (15.82+33.51)/2=24.67%. In 2/5 (40%) of the active lesions, MOG co-localization is >25%. Scale bars represent in b 2.5mm, in c and e 100µm and in d and f 10µm.

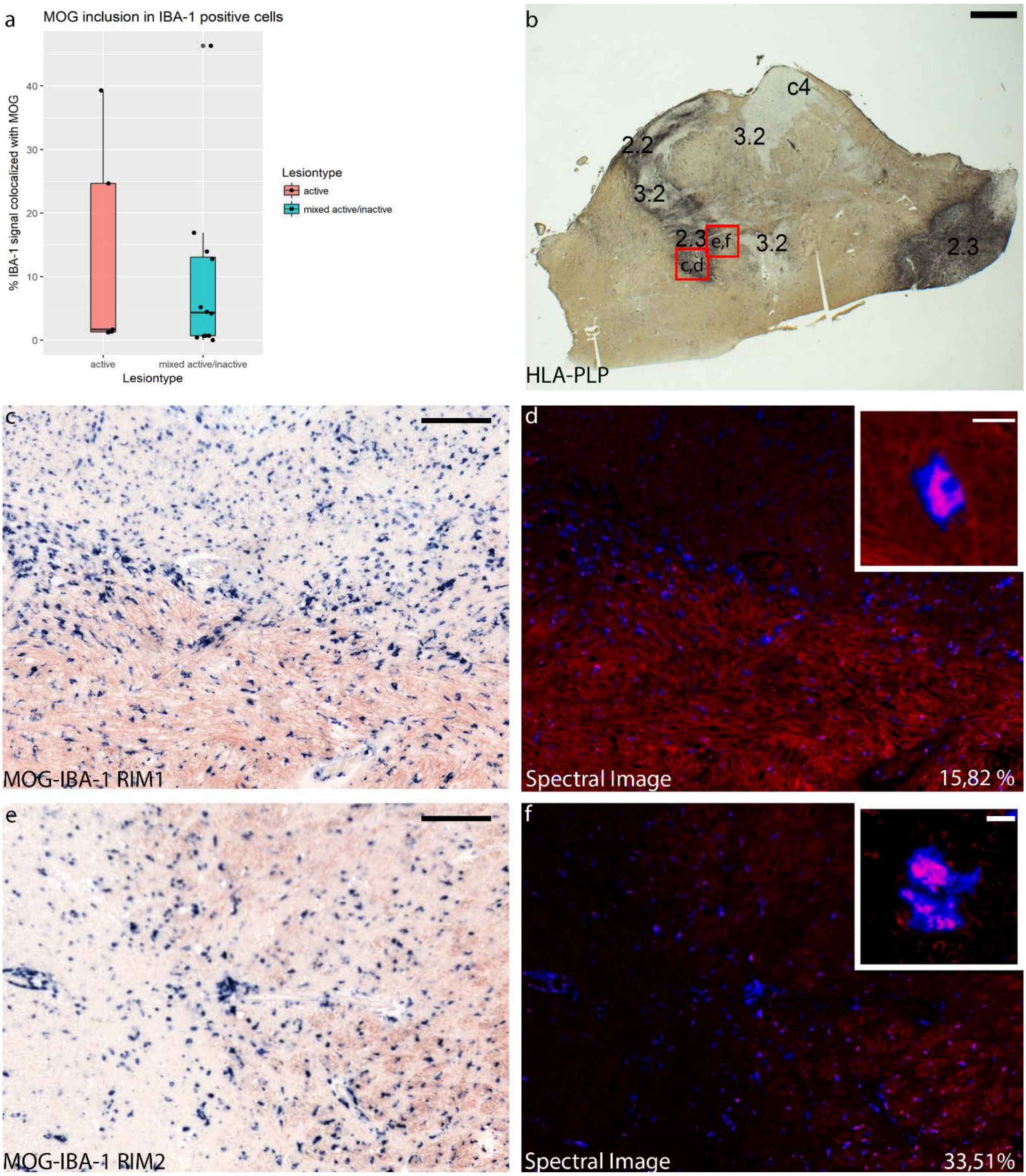

Article title: Progressive Multiple Sclerosis patients show substantial lesion activity that correlates with clinical disease severity and sex: a retrospective autopsy cohort analysis

Journal name: Acta Neuropathologica

Author names: Sabina Luchetti# MD PhD, Nina L. Fransen# MD MSc, Corbert G. van Eden PhD, Valeria Ramaglia PhD, Matthew Mason* PhD, Inge Huitinga* PhD

Corresponding author: Inge Huitinga, PhD, Leader Neuroimmunology group Netherlands Institute for Neuroscience, e-mail i.huitinga@nin.knaw.nl
