# Supplementary material for: Progressive multiple sclerosis patients show substantial lesion activity that correlates with clinical disease severity and sex: a retrospective autopsy cohort analysis
Source: Acta Neuropathol. 2018 Feb 13;135(4):511–28. doi: 10.1007/s00401-018-1818-y (PMC5978927; doi:10.1007/s00401-018-1818-y)
Supplement: Supplementary file 4 — Supplementary material 4 (PDF 203 kb) [file 401_2018_1818_MOESM4_ESM.pdf]

Online resource 4:

Supplemental Figure 3 Relationship between the proportion of lesion type and disease severity

a. Lesion type proportions versus years to EDSS-6. Cases have been grouped by years to EDSS-6 as follows: [0,6] n=32; (6,9] n=27; (9,13] n=26; (13,18] n=29; (18, 27.2] n=26; (27.2,56] n=28. b. Lesion type proportions versus years to EDSS-8. Cases have been grouped by years to EDSS-8 as follows: [3,12.3] n=23; (12.3,17] n=25; (17,21] n=21; (21,26] n=25; (26,35] n=19; (35,59] n=22. c. Lesion type proportions versus duration of disease. Cases have been grouped by duration of disease as follows: [2,16] n=32; (16,22] n=32; (22,26] n=24; (26,32] n=30; (32,42] n=27; (42,64] n=27. Mixed active/inactive lesions and lesions with foamy microglia/macrophages are overrepresented in patients with shorter time to EDSS-6 and shorter disease duration, while proportions of inactive and remyelinated lesions are overrepresented in patients with longer time to EDSS-6 and longer disease duration.

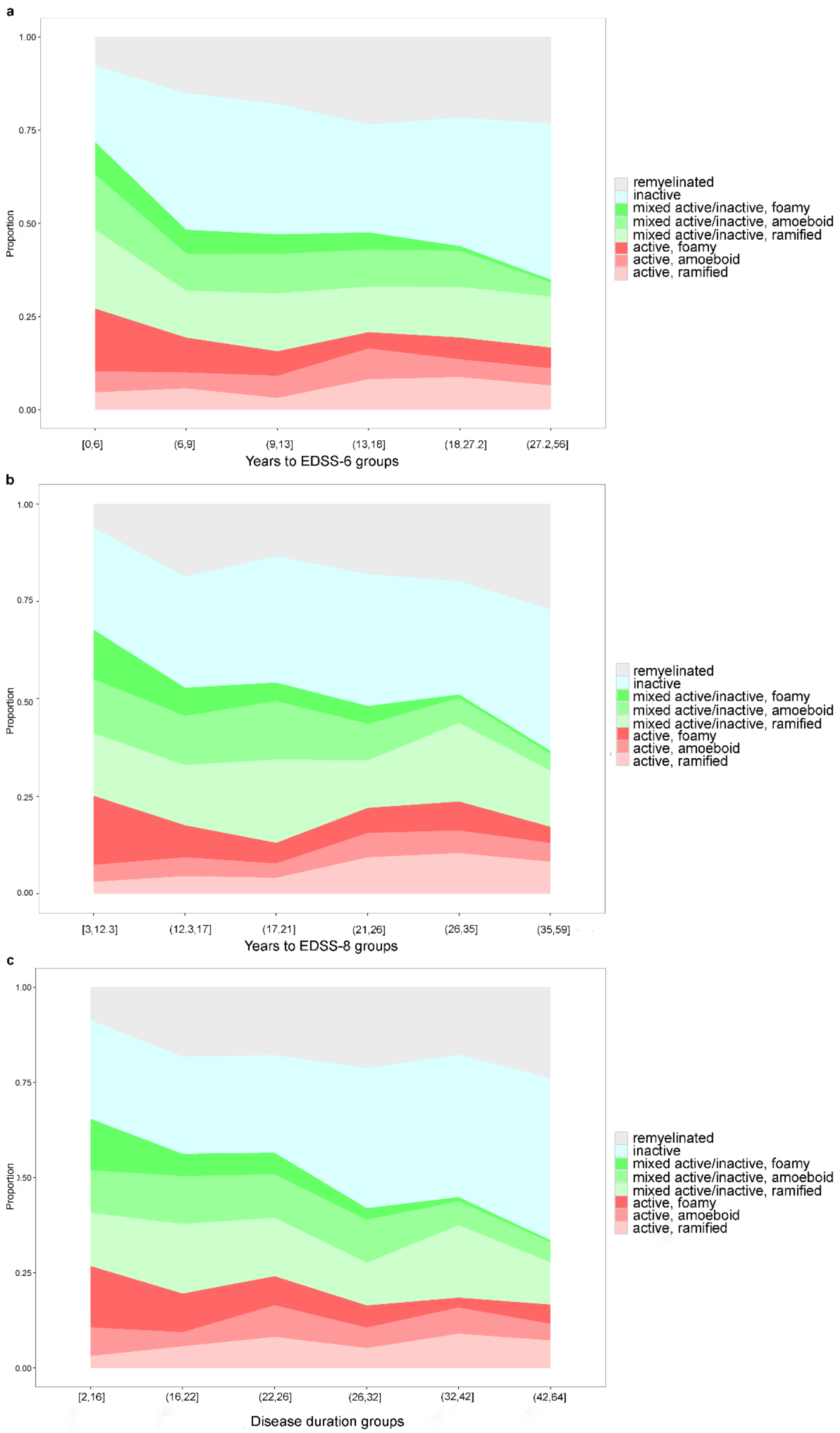

a
1.00
0.75
0.50
0.25
0.00
Proportion
[0,6]
(6,9]
(9,13]
(13,18]
(18,27.2]
(27.2,56]
Years to EDSS-6 groups
remyelinated
inactive
mixed active/inactive, foamy
mixed active/inactive, amoeboid
mixed active/inactive, ramified
active, foamy
active, amoeboid
active, ramified
b
1.00
0.75
0.50
0.25
0.00
Proportion
[3,12.3]
(12.3,17]
(17,21]
(21,26]
(26,35]
(35,59]
Years to EDSS-8 groups
remyelinated
inactive
mixed active/inactive, foamy
mixed active/inactive, amoeboid
mixed active/inactive, ramified
active, foamy
active, amoeboid
active, ramified
c
1.00
0.75
0.50
0.25
0.00
Proportion
[2,16]
(16,22]
(22,26]
(26,32]
(32,42]
(42,64]
Disease duration groups
remyelinated
inactive
mixed active/inactive, foamy
mixed active/inactive, amoeboid
mixed active/inactive, ramified
active, foamy
active, amoeboid
active, ramified

Article title: Progressive Multiple Sclerosis patients show substantial lesion activity that correlates with clinical disease severity and sex: a retrospective autopsy cohort analysis

Journal name: Acta Neuropathologica

Author names: Sabina Luchetti# MD PhD, Nina L. Fransen# MD MSc, Corbert G. van Eden PhD, Valeria Ramaglia PhD, Matthew Mason* PhD, Inge Huitinga* PhD

Corresponding author: Inge Huitinga, PhD, Leader Neuroimmunology group Netherlands Institute for Neuroscience, e-mail **i.huitinga@nin.knaw.nl**,
