# Supplementary material for: Progressive multiple sclerosis patients show substantial lesion activity that correlates with clinical disease severity and sex: a retrospective autopsy cohort analysis
Source: Acta Neuropathol. 2018 Feb 13;135(4):511–28. doi: 10.1007/s00401-018-1818-y (PMC5978927; doi:10.1007/s00401-018-1818-y)
Supplement: Supplementary file 5 — Supplementary material 5 (PDF 181 kb) [file 401_2018_1818_MOESM5_ESM.pdf]

Online resource 5:

Supplemental Figure 4 Relationship between cortical grey matter lesions, sex and time to EDSS-6 in years

a. An inverse relationship was found between grey matter lesion presence and severity in females (GLM, p=0.004) but not in males (p = 0.56). Patients are grouped based on years to EDSS-6 and number of patients per group is shown in the bar. b. Females with cortical grey matter lesions have higher disease severity (p=0.004, students t-test), whereas in males no significant difference was found (p=0.730, students t-test).

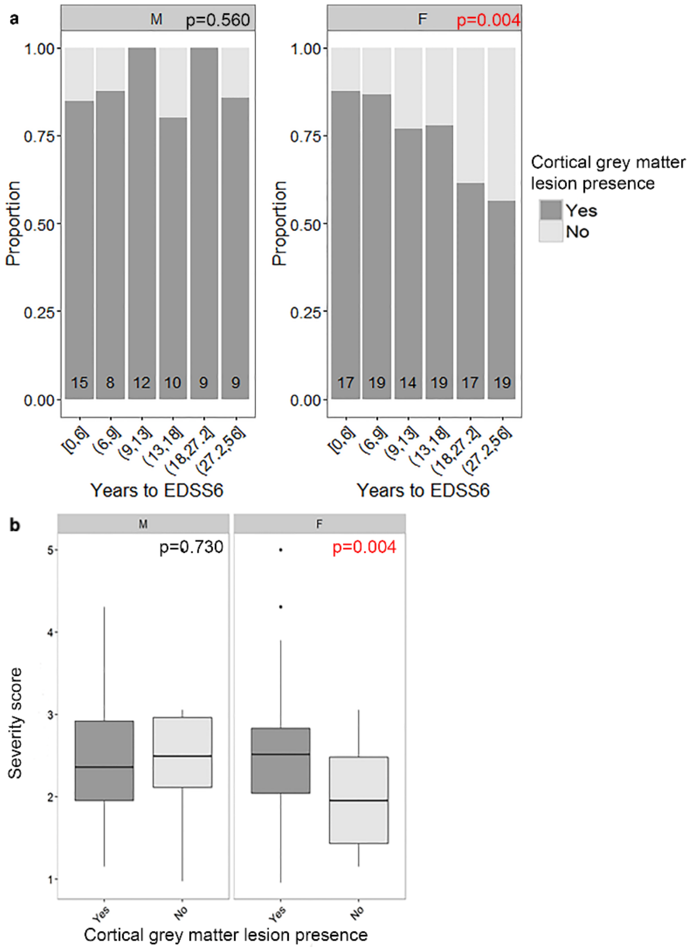

Article title: Progressive Multiple Sclerosis patients show substantial lesion activity that correlates with clinical disease severity and sex: a retrospective autopsy cohort analysis

Journal name: Acta Neuropathologica

Author names: Sabina Luchetti# MD PhD, Nina L. Fransen# MD MSc, Corbert G. van Eden PhD, Valeria Ramaglia PhD, Matthew Mason* PhD, Inge Huitinga* PhD

Corresponding author: Inge Huitinga, PhD, Leader Neuroimmunology group Netherlands Institute for Neuroscience, e-mail i.huitinga@nin.knaw.nl
