# Supplementary material for: Progressive multiple sclerosis patients show substantial lesion activity that correlates with clinical disease severity and sex: a retrospective autopsy cohort analysis
Source: Acta Neuropathol. 2018 Feb 13;135(4):511–28. doi: 10.1007/s00401-018-1818-y (PMC5978927; doi:10.1007/s00401-018-1818-y)
Supplement: Supplementary file 6 — Supplementary material 6 (PDF 168 kb) [file 401_2018_1818_MOESM6_ESM.pdf]

Online resource 6:

Supplemental Figure 5 Relationship between cortical grey matter lesion subtypes and lesion activity

The presence of leukocortical (type I) and intracortical (type II) lesions is strongly related to lesion load, reactive load, proportions of mixed active/inactive lesions and remyelinated lesions, while subpial III and IV lesions show only a relation with lesion load. Student t-test with unequal variances is used.

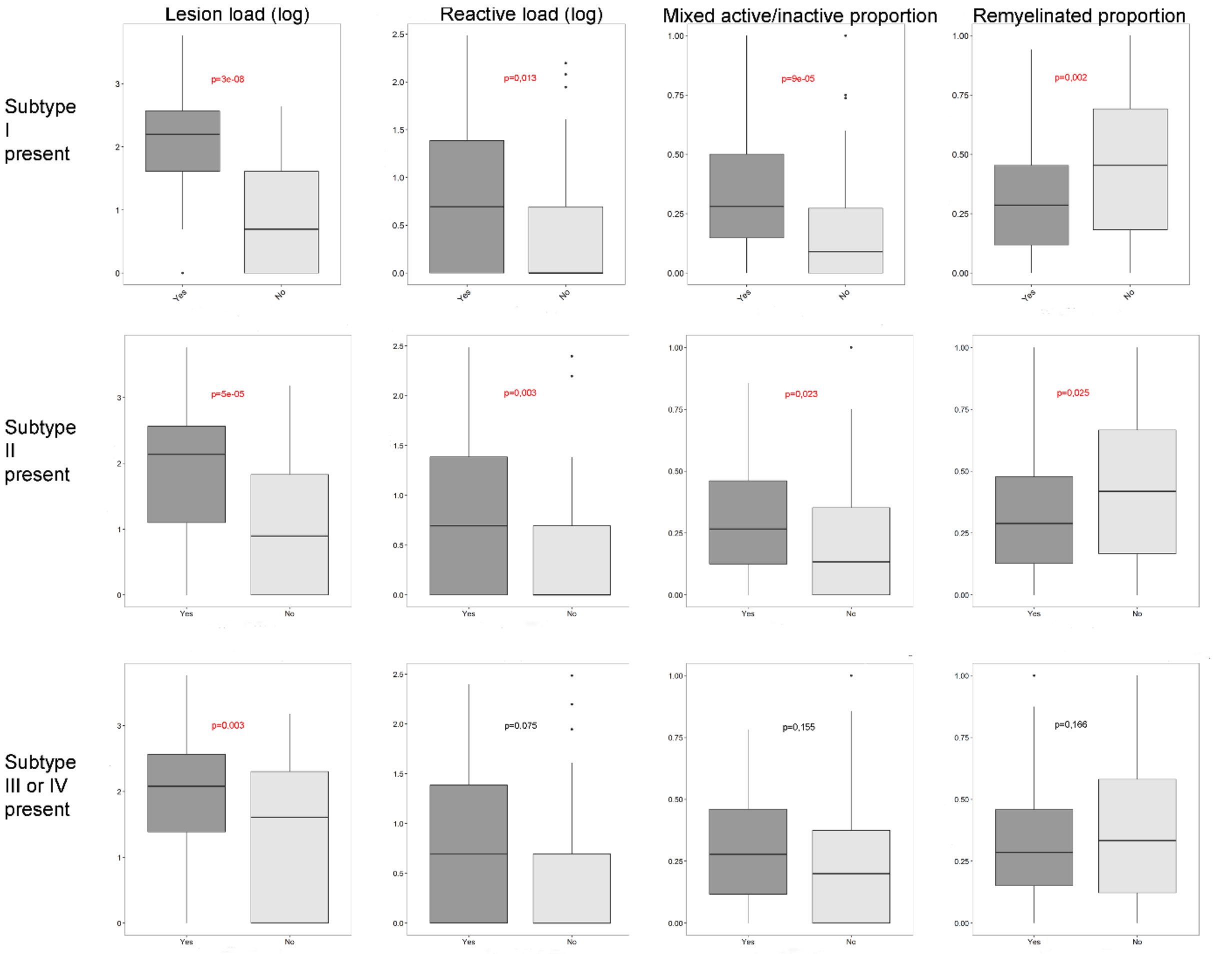

Article title: Progressive Multiple Sclerosis patients show substantial lesion activity that correlates with clinical disease severity and sex: a retrospective autopsy cohort analysis

Journal name: Acta Neuropathologica

Author names: Sabina Luchetti# MD PhD, Nina L. Fransen# MD MSc, Corbert G. van Eden PhD, Valeria Ramaglia PhD, Matthew Mason* PhD, Inge Huitinga* PhD

Corresponding author: Inge Huitinga, PhD, Leader Neuroimmunology group Netherlands Institute for Neuroscience, e-mail i.huitinga@nin.knaw.nl
