# Supplementary material for: Progressive multiple sclerosis patients show substantial lesion activity that correlates with clinical disease severity and sex: a retrospective autopsy cohort analysis
Source: Acta Neuropathol. 2018 Feb 13;135(4):511–28. doi: 10.1007/s00401-018-1818-y (PMC5978927; doi:10.1007/s00401-018-1818-y)
Supplement: Supplementary file 7 — Supplementary material 7 (PDF 116 kb) [file 401_2018_1818_MOESM7_ESM.pdf]

Online resource 7:

Supplemental Figure 6 Lesion type proportion by sampled location

Spinal cord and brainstem are both standardly sampled, showing spinal cord has a higher percentage of inactive and remyelinated lesions compared to brainstem. PLA/MRI are not standardly sampled and have a significantly lower proportion of active and mixed active/inactive lesions and a higher proportion of remyelinated lesions, compared to brainstem (quasibinomial generalized linear model).

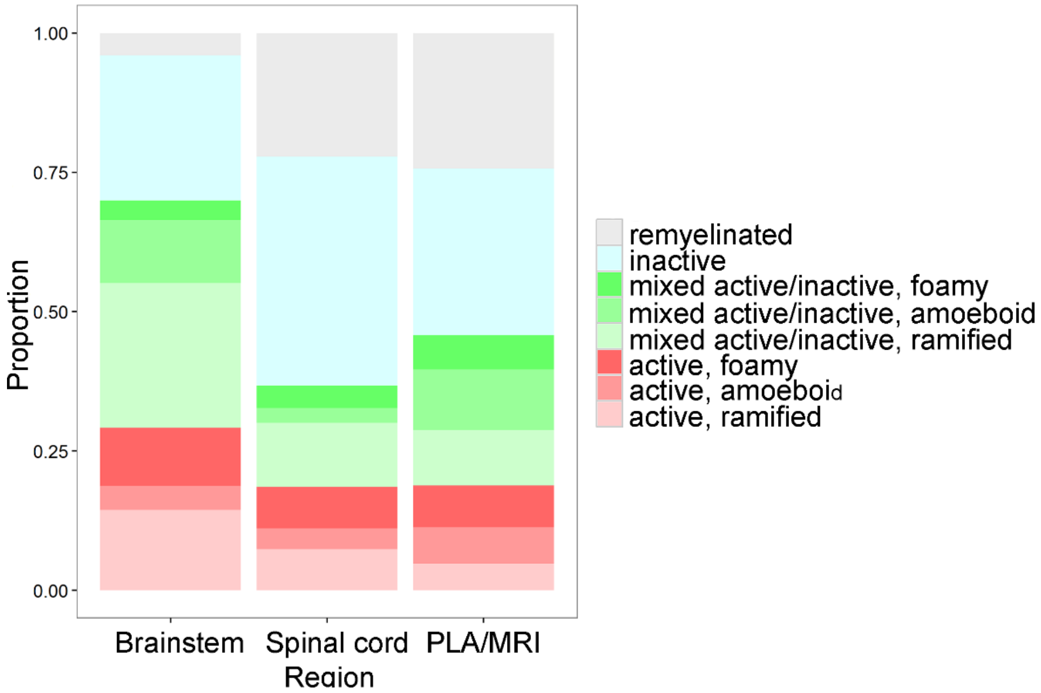

Article title: Progressive Multiple Sclerosis patients show substantial lesion activity that correlates with clinical disease severity and sex: a retrospective autopsy cohort analysis

Journal name: Acta Neuropathologica

Author names: Sabina Luchetti# MD PhD, Nina L. Fransen# MD MSc, Corbert G. van Eden PhD, Valeria Ramaglia PhD, Matthew Mason* PhD, Inge Huitinga* PhD

Corresponding author: Inge Huitinga, PhD, Leader Neuroimmunology group Netherlands Institute for Neuroscience, e-mail i.huitinga@nin.knaw.nl,
